# Experiences with telemedicine-based follow-up of chronic conditions: the views of patients and health personnel enrolled in a pragmatic randomized controlled trial

**DOI:** 10.1186/s12913-024-10732-7

**Published:** 2024-03-14

**Authors:** Susanna Sten-Gahmberg, Kine Pedersen, Ingrid Gaarder Harsheim, Hanna Isabel Løyland, Birgit Abelsen

**Affiliations:** 1 Oslo Economics, Klingenberggata 7, Oslo, 0161 Norway; 2 Present Address: The Finnish Centre for Pensions, Eläketurvakeskus, FI-00065 Finland; 3 Department of Health Management and Health Economics, University of Oslo, Postboks 1089, Blindern, Oslo, 0317 Norway; 4 Department of Community Health, UiT - The Arctic University of Norway, Tromsø, 9037 Norway

**Keywords:** Primary healthcare, Telemedicine, Chronic disease, Patient experience, Individual treatment plan, Health personnel, Process evaluation

## Abstract

**Background:**

Telemedicine is often promoted as a possible solution to some of the challenges healthcare systems in many countries face, and an increasing number of studies evaluate the clinical effects. So far, the studies show varying results. Less attention has been paid to systemic factors, such as the context, implementation, and mechanisms of these interventions.

**Methods:**

This study evaluates the experiences of patients and health personnel enrolled in a pragmatic randomized controlled trial comparing telemedicine-based follow-up of chronic conditions with usual care. Patients in the intervention group received an individual treatment plan together with computer tablets and home telemonitoring devices to report point-of-care measurements, e.g., blood pressure, blood glucose or oxygen saturation, and to respond to health related questions reported to a follow-up service. In response to abnormal measurement results, a follow-up service nurse would contact the patient and consider relevant actions. We conducted 49 interviews with patients and 77 interviews with health personnel and managers at the local centers. The interview data were analyzed using thematic analysis and based on recommendations for conducting process evaluation, considering three core aspects within the process of delivering a complex intervention: (1) context, (2) implementation, and (3) mechanisms of impact.

**Results:**

Patients were mainly satisfied with the telemedicine-based service, and experienced increased safety and understanding of their symptoms and illness. Implementation of the service does, however, require dedicated resources over time. Slow adjustment of other healthcare providers may have contributed to the absence of reductions in the use of specialized healthcare and general practitioner (GP) services. An evident advantage of the service is its flexibility, yet this may also challenge cost-efficiency of the intervention.

**Conclusions:**

The implementation of a telemedicine-based service in primary healthcare is a complex process that is sensitive to contextual factors and that requires time and dedicated resources to ensure successful implementation.

**Trial registration:**

The trial was registered in www.clinicaltrials.gov (NCT04142710). Study start: 2019-02-09, Study completion: 2021-06-30, Study type: Interventional, Intervention/treatment: Telemedicine tablet and tools to perform measurements. Informed and documented consent was obtained from all subjects and next of kin participating in the study.

**Supplementary Information:**

The online version contains supplementary material available at 10.1186/s12913-024-10732-7.

## Background

Telemedicine is often promoted as a tool that could be used to solve some of the challenges faced by healthcare systems in many countries. Among other things, telemedicine could improve access to healthcare services, improve the quality of follow-up of persons with for example chronic disease, and improve patient satisfaction by allowing patients to take a more active role in the follow-up of their health. Telemedicine could furthermore be associated with a lower cost than regular follow-up, particularly by reducing the use of more costly services [1, 2].

However, evaluations of telemedicine-based interventions show mixed results. In general, telemedicine interventions tend to result in improved patient experience. Leonardsen et al. [3] summarized empirical studies exploring patient experiences with telemedicine interventions. The studies found that patients feel more empowered, learn more about their condition (health literacy), increase their awareness of symptoms and treatment, and feel safer and more self-efficient. Therefore, telemedicine has the potential to improve self-efficacy and independence through patient involvement. However, several factors hinder widespread use of telemedicine, including technology barriers, lack of computer literacy, insufficient of lacking of financial incentives, human inertia, and organizational and cultural issues in healthcare organizations [4–8].

Empirical studies on the effectiveness of telemedicine interventions tend to vary more in their findings. The effects of interventions appear to differ between clinical contexts [9]. For instance, one meta-analysis found that telemedicine interventions can significantly reduce HbA1c levels among patients with diabetes, while other studies reported no change or an increase in HbA1c [10]. Another review found that telemedicine interventions reduced chronic obstructive pulmonary disease (COPD) exacerbations in some studies, but not all [11]. Cost-effectiveness also vary across studies; while some find evidence of reduced resource use, e.g., through fewer hospital admissions, others find no such evidence [12–14].

The heterogeneity in outcomes of different interventions observed in individual studies and meta-analyses is mainly due to differences in type of technology and patients under study. Furthermore, telemedicine studies vary with respect to type of involved personnel, and some controlled studies lack information about patients in control groups [15]. As primary healthcare services vary with respect to content, organization and quality across countries, generalizability of studies may be limited. Flumignan and colleagues [16] argue that there is currently insufficient evidence to determine the effectiveness of telemedicine interventions for different patient population sand settings, and whether such interventions can replace standard face-to-face consultations. They suggest that more randomized trials are needed to increase the level of evidence and reduce potential bias and confounding factors.

Due to variation in both the intervention and the context of implementation, telemedicine interventions can be considered to be complex interventions. The impact of telemedicine-based interventions can thus be analyzed using a framework of complex interventions [17–19]. According to Skivington and co-authors [19], complex intervention research goes beyond asking whether an intervention works in the sense of achieving its intended outcome, to exploring more broadly for example what other impact it has, how it works, how it interacts with the context in which it is implemented, and how the evidence can be used to support real world decision making. This approach is broader compared to traditional evaluation of interventions in medical and social sciences, yet comes at a trade-off between more precise unbiased answers to narrow questions and more uncertain answers to broader, more complex questions when comparing these approaches.

We have conducted a pragmatic randomized controlled trial of a telemedicine-based intervention in Norway. The primary aim was to explore a telemedicine-based intervention in follow-up of patients with chronic health conditions within a primary care setting, in terms of clinical effectiveness, resource use, and real-life implementation challenges. Documentation of the trial and results from the effectiveness analysis are reported elsewhere [20, 21]. In Norway, municipalities are responsible for primary care services, while the state is responsible for the specialist healthcare services provided mainly by hospitals. Patients with chronic conditions typically attend regular follow-up consultations at their GP. In addition, some patients receive home-based services and some attend check-ups at the hospital. Inspired by frameworks for evaluating complex interventions [17–19], we conducted a process evaluation of the same trial to better understand the context in which the intervention was implemented, practical implementation, as well as barriers and promoting factors. The results from the process evaluation are presented in this article. The analysis relies on in-depth interviews and surveys with patients, next of kin and health personnel, conducted at different stages in the trial.

This study contributes both to the empirical literature on the impact of telemedicine-based interventions and to the literature on process evaluations of complex interventions. First, it enhances our understanding of how to implement a telemedicine-based intervention in practice, including aspects such as organization and interaction with other services. Second, it describes the experiences of both patients who receive the intervention, health personnel who provide the intervention, and health personnel who interact with the intervention through their follow-up of patients. Consequently, it points towards several ways in which the intervention can lead to change or meet barriers and can thus be used as guidance for real-life implementation. Third, while the use of process evaluations as part of pragmatic trials has been stressed in the literature, the body of literature providing such information is still scarce. This study gives an example of how such a process evaluation can be conducted, and how it can give additional insight to the evaluation of randomized controlled trials.

## Methods

### Process evaluation

To better understand the local implementation of the telemedicine-based intervention and its impact on patients and healthcare services, we conducted a process evaluation as part of a pragmatic randomized controlled trial [22]. In this exploratory analysis, we relied on semi-structured interviews with key actors in the different healthcare contexts and a convenience sample of patients and their next of kin. Informants were recruited with the help of staff at the local follow-up service. Follow-up service nurses and local project managers were interviewed multiple times during the trial, while patients, their next of kin, and health personnel were mainly interviewed once, either shortly after inclusion or towards the end of the trial. The CONSORT 2010 checklist and the COREQ reporting guidelines were used when developing this manuscript.

### Trial design

The trial was designed as a pragmatic, non-blinded, multi-center, individual, randomized-controlled trial at six local centers spread across six municipalities in Norway, and has been described in detail elsewhere [20, 21]. In brief, the Norwegian Directorate of Health (NDH) was the principal for the trial and provided guidelines for the implementation of the intervention. The purpose of the trial was to obtain knowledge about the consequences of telemedicine-based patient follow-up compared to usual primary care follow-up for patients with chronic health conditions.

Patients eligible for the trial included adult patients with at least one chronic condition, a considerable disease burden and comprehensive medical needs as judged by health personnel, and a medium to high risk of worsening of their health condition, hospitalization, or increased need for medical and care services. Patients were recruited to the trial from February 19th, 2019, through June 30th, 2020. The follow-up period of participants was 12–18 months depending on the date of inclusion. Randomized inclusion of patients to the trial ended on March 17th, 2020, due to uncertainty related to the Covid-19 pandemic. From this day, all eligible patients were included in the study in a non-randomized intervention group. 735 patients were included in the trial, of whom 5 withdrew their consent to participate after inclusion. Of the remaining 730 participants, 261 participants were randomized into the intervention group and 276 to the control group. 193 participants were included in the non-randomized intervention group.

Outcomes were assessed using mixed methods through three main analyses: (1) effectiveness analysis aimed at measuring outcomes in terms of patients’ health status, user experience and resource utilization in the healthcare system; (2) process evaluation aimed at studying aspects of context, implementation, and mechanisms of impact; and (3) cost-benefit analysis aimed at evaluating the societal value of the telemedicine-based follow-up compared to usual care. The results from the process evaluation are presented in this article, while the results from the effectiveness and the cost-benefit analyses are reported in Sten-Gahmberg et al. [21] and briefly summed up in the Discussion section.

### Telemedicine-based intervention

#### Organization and responsibilities

**Fig. 1:**
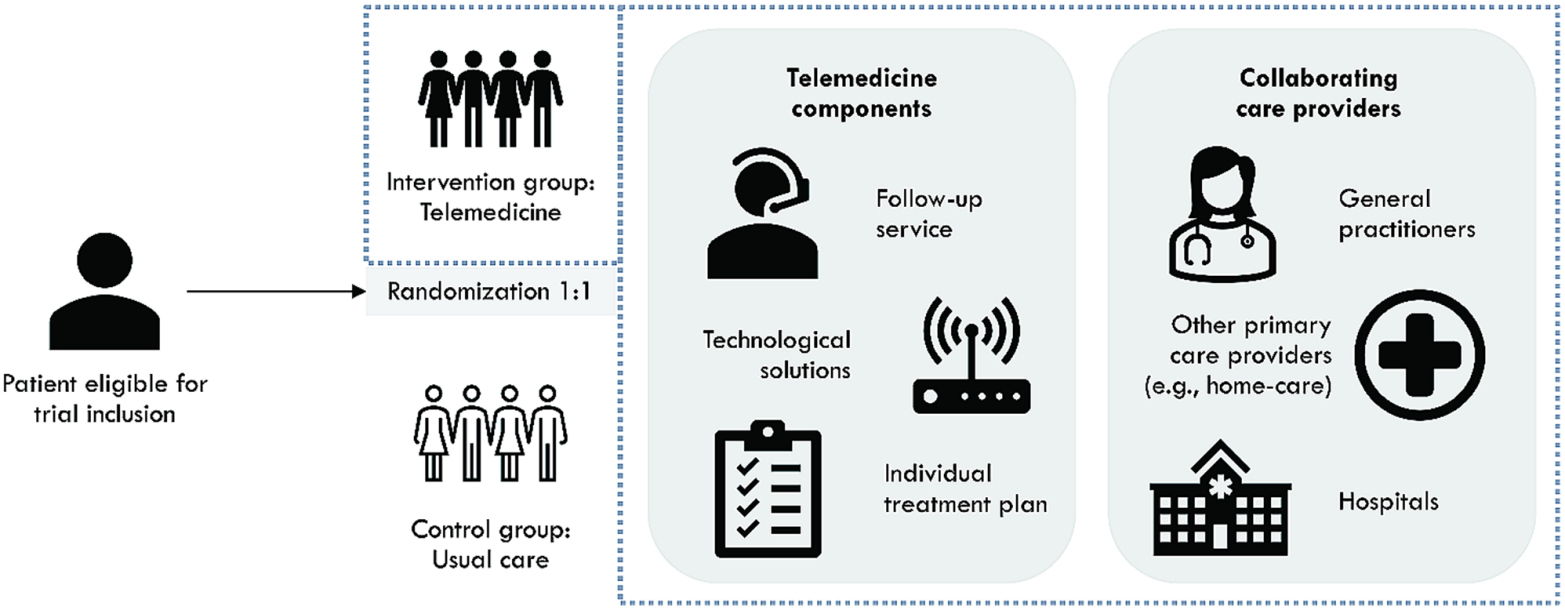
Overview of the telemedicine-based intervention

The NDH outlined guidelines for the organization of the telemedicine-based intervention, yet the six local centers could adjust the telemedicine-based intervention according to local context and needs (Fig. 1). The centers were responsible for recruiting patients to the study, providing the intervention, and facilitating cooperation between GPs, the follow-up service, other primary health and care services and hospitals. The local centers were also responsible for technical equipment and software, including procurement, logistics, training in the use of equipment and user support. Three different suppliers of technological solutions were involved. In addition, the centers differed with respect to their target population (main diagnoses and disease burden), recruitment channels, and the design of the intervention itself.

The NDH expected service development at the local centers during the study period, to inform future service design and implementation. This led to variations in the composition of the intervention and in the study population, both across the centers and within each center over time.

#### Telemedicine-based follow-up

Participants who received telemedicine-based follow-up, received a computer tablet and relevant home monitoring equipment, and were offered user training. The tablet was used to answer simple questions about the patient’s own health and/or to monitor measurements related to health status (such as blood pressure, blood sugar, oxygen saturation or weight). The measurements were automatically transferred to the patient’s tablet and to the follow-up service.

After an initial start-up period where the follow-up service became familiar with the habitual state of the patient, a follow-up service nurse, the patient, and the GP prepared an individual treatment plan based on the patient’s goals, disease burden and risk of deterioration. The treatment plan was based on a traffic light model, outlining different medicinal and non-medicinal actions that the patient could take depending on whether their measurements were in a habitual state (green), slightly (yellow) or significantly (red) deviating from the habitual state. The threshold values for abnormal measurements were set by the health personnel in consultation with the patient.

The follow-up service and the patient agreed on how often the patient should carry out measurements and answer questions, which depended on the patient’s health condition and diagnoses, as well as individual preferences. Most patients registered measurements daily, but the frequency could also vary over time. The technological solutions informed the patient and the follow-up service when the measurement results deviated from the patient’s normal values. The nurse responded to abnormal results, and considered, in consultation with the patient, whether the patient should contact the GP or the emergency room. If a patient did not report the scheduled measurements, this could also prompt the follow-up service to contact the patient. Measurements within the patient’s normal range did not prompt any action from the follow-up service.

#### Control group follow-up

Participants in the control group received usual clinical care. The specific services provided as part of usual clinical care varied according to their health condition and needs. Most patients in the control group were followed up by their GP.

#### Participants and data collection

The data collection of this study, including a description of the participants, the type of data, the themes and year of data collection, is presented in Table 1 and further described in the following text.

**Table 1:** Overview of the data production

Type of information	Participants	Year	Number of participants
Patient and next of kin experience, sense of security, coping, health status, time-use, shift of responsibility and tasks	Patient	2019	23
		2021	25
	Next of kin	2019	11
		2021	8
Recruitment, inclusion and exclusion criteria, organization, follow-up of patients, collaboration with other healthcare providers, costs and benefits, success criteria and challenges for telemedicine follow-up	Local centre administration	2019	13
		2020	12
		2021	10
	Follow-up service nurse	2019	11
	GP	2019	10
		2021	6
	Hospital and primary healthcare staff	2020	4
		2021	11

#### Patients and their next of kin

Patients who received telemedicine-based follow-up were recruited to the interview study with the help of the local project staff at each local center. In 2019, local staff were asked to recruit 2–3 patients, in addition to 2–3 patients with their next of kin. In 2021, the staff were asked to recruit 2–3 patients, of which 1–2 were to be interviewed together with their next of kin. We do not know if any patients refused to participate. However, some of the patients were unable to participate in an interview themselves, e.g., because of their health condition, but were represented in the interview by their next of kin. Thus, these interviews secured insights into the views of patients who we could not interview directly. Including these patients, we interviewed a total of 48 patients either in the fall of 2019 or in the spring of 2021.

We encouraged recruitment of patients with varying backgrounds (e.g., health conditions) and experience with follow-up. We also asked the staff not only to recruit the patients they had the closest contact with or those they knew had particularly positive experiences with the project. In 2021, we specifically asked to interview individuals who were not interviewed in 2019 to obtain experiences from different individuals.

In total, 19 next of kin participated in the interviews. The next of kin was typically the spouse of the patient, but children were also represented, and the majority were female. In the cases where we interviewed both a patient and their next of kin, both parties were present during the entire interview. Some questions were specifically directed towards either the patient or the next of kin, yet both could complement and comment on each other.

The purpose of the interviews with patients and their next of kin was to collect data about their experience with telemedicine-based follow-up and different aspects of the patient follow-up (see Additional file 1 and 2). The interviews lasted between 15 and 60 min, typically around 45 min. The interviews were conducted either at the patients’ homes, in municipal facilities or via telephone or video call. Most of the phone or video call interviews were conducted in 2021, because of the ongoing pandemic. In total, approximately half of the interviews were conducted face-to-face.

Most patients were recruited to the trial because of their COPD diagnosis, but there were also several patients with cardiovascular diseases, diabetes and cancer. Patients recruited to the interview study had a similar distribution of diagnoses. About two thirds of the interviewed patients were male. Patient age was not registered in the interview material, but most of the patients were in their late sixties or older while a handful of the interviewed patients were younger. Reassuringly, the age of the interviewed patients fit well with the age of the total population included in the study [21].

#### Center staff and follow-up service nurses

The purpose of the interviews with the project staff was to gain insight in the organization of the service, collaboration, and interaction with other healthcare services and their experience with telemedicine-based follow-up (see Additional file 3). The six local centers were led by a project manager or a management team, depending on the local organization of the follow-up service. Most of the project managers held a degree in nursing, but some held degrees in occupational therapy or sociology. Some had further education or a master’s degree in health informatics, law, advanced clinical nursing, or welfare technology. In five of the six local centers we interviewed the whole management team together. The sixth center, which was also the largest, was divided into four subcenters in different parts of a larger municipality. The center managers of these four subprojects were interviewed individually. We interviewed the center management three times during the trial period. The first round of interviews was conducted at each of the local follow-up service, except from one, which was conducted by video call. The remaining rounds of interviews were conducted by video call.

The purpose of the interviews with follow-up service nurses was to gain information of the patient groups receiving telemedicine-based follow-up, how they followed-up, collaboration and interaction with other healthcare services and their experience with telemedicine-based follow-up (see Additional file 4). The follow-up services were run by two or more nurses depending on the local organization. The majority held a degree in nursing. Most had a bachelor level degree, and a minority had a master level degree. We conducted one round of interviews with follow-up service nurses at the local centers in 2019 (except from one interview which was conducted by video call).

Throughout the project period, the NDH arranged several meetings and workshops for both project management and follow-up service nurses. These workshops were used to discuss different topics related to the telemedicine follow-up, such as identification and inclusion of patients, telemedicine follow-up, and collaboration with other healthcare service providers. Our notes from these workshops have also been analyzed as part of this study.

#### Other health personnel

GPs were to approve their patients’ participation in the trial. In collaboration with the patient and the follow-up service nurse, they also took part in the development of the individual treatment plan of their patients. In some municipalities, GPs could follow the measurements of their patients using the software from the provider of the technological solution. To investigate the experiences of the GPs with the intervention, we conducted two rounds of interviews, in 2019 and 2021 (see Additional file 5).

Furthermore, some GPs also took part in NDH’s workshops throughout the project period, and information collected through these workshops are part of the material analyzed in this study.

We have also interviewed hospital and primary healthcare staff about their collaboration with the follow-up service and their follow-up of patients in the trial (see Additional file 6 and 7). In hospitals, these informants were mainly nurses at a pulmonary medicine or cardiology department, with responsibility for identifying patients who would be suitable for participation in the trial. Primary healthcare staff included nurses in home-based care services (HCS) and at the municipal service allocation office.

The total number of interviews was a result of time and resource constraints. Initially, we intended to conduct a somewhat higher number of interviews, but it proved challenging for the project managers to recruit participants (patients and health personnel). However, our impression after interviewing the participants they managed to recruit, was that conducting more interviews would provide limited new information, indicating that we had reached a point of saturation in the interview study.

All authors are well experienced interviewers, and all participated in conducting the interviews. The interviewers got to know the local projects and project managers relatively well since they were interviewed repeatedly, and they met in various contexts related to project implementation. There were no similar relationships with the other informants. The participants were provided with pilot tested interview guides prior to the interviews and given written information about the purpose of the data collection and their privacy rights as study participants. The interview guides are listed as Electronic supplementary materials. Informed and signed consent was obtained from all subjects and next of kin participating in the study. Due to pandemic restrictions and some of the interviews being conducted by phone or video call, a small group of next of kin gave verbal consent to participation, which was recorded on tape.

### Analysis

All interviews were recorded. Interviews conducted in 2019 were transcribed, while we took detailed notes from subsequent interviews. Transcripts were not returned to or commented on by the participants. The interview data and our other notes were analyzed manually, using thematic analysis [23]. The steps included familiarizing with the data, generating initial codes, further categorization of the coded data, searching for themes, reviewing themes, defining, as well as naming themes, and the write-up. The analysis was analyst-driven as it was based on guidance on process evaluation [17], stressing three core aspects within the process of delivering a complex intervention. These were our main themes: (1) *context*, i.e., the environment in which the intervention was rolled out and how this may have affected the implementation and the impact of the intervention, (2) *implementation*, i.e., how the intervention was designed and rolled out in practice, whether implementation was in line with the plan, and whether there were changes of the intervention in the study period, and (3) *mechanisms of impact*, i.e., patient and health personnel responses and interactions with the intervention, and ways in which the intervention may have contributed to the observed outcomes. The sub-themes and further categories that emerged in the analysis were arranged under these main themes. Table 2 details the main themes, sub-themes, and categories. Some participant quotations are presented in the result section to illustrate the findings. The participants did not provide feedback on the findings.

**Table 2:** Main themes, sub-themes, and categories from the thematic analysis

Main theme	Sub-theme	Category
1 Context	1a Characteristics of the participating municipalities	
	1b Collaboration with local hospitals	
	1c The GP service	
	1d The Covid-19 pandemic	
2 Implementation	2a Organization of the follow-up service and integration with other services	
	2b Inclusion of patients	
	2c Follow-up of patients	
3 Mechanisms of impact	3a Patient interaction with the service	3a1 User interaction
		3a2 User satisfaction
	3b Healthcare staff responses to the service	3b1 Follow-up service responses
		3b2 Municipal home-based care services (HCS)
		3b3 GP responses
		3b4 Specialized healthcare

### Ethics

The Regional Committee for Medical and Health Research Ethics assessed the study protocol (REC south-east A, 2018/1927). A Data Protection Impact Assessment, conducted together with the Norwegian Centre for Research Data in January 2019, concluded that the data collection and storage were conducted in accordance with the GDPR (988680). The protocol was registered in www.clinicaltrials.gov (NCT04142710). All study participants signed a consent form upon invitation to participate in the study.

## Results

The presentation of the results of the study is divided into three main themes: (1) *context*, (2) *implementation*, and (3) *mechanisms of impact*. In the following text, we present our findings related to the respective theme.

### 1: Context

The purpose of this part of the analysis is to describe the context in which the trial was conducted. This is important for understanding the background for piloting the telemedicine-based service, but also for understanding choices related to the local implementation, and factors that may have affected the outcomes of the service.

#### 1a: Characteristics of the participating municipalities

While the NDH was the principal for the trial and provided guidelines for the implementation, the local centers were run by the participating municipalities, which due to self-governance can decide how healthcare is organized on the local level within the frames of current laws and regulations. As the municipalities are different with regards to geographic size, population and density, organization of other municipal health and care services and previous experience with telemedicine, the freedom to adapt the service to the local context was key to develop a service that was well-suited to each municipality. In consequence, the local centers varied in organization of the follow-up service, which patient groups they prioritized and how frequently the patients were followed up.

Local circumstances played a significant role with respect to potential challenges faced in the trial. In the larger municipalities (i.e., population size), where there were many GPs and more home-based care service (HCS) units, it was challenging to inform all the relevant people about the trial. Local circumstances could also work in favor of successful implementation of telemedicine-based follow-up. In some scarcely populated municipalities, the driving distance to patients’ home could be up to several hours, and thus telemedicine could reduce the travel time for the HCS considerably, which in turn could alleviate the shortage of health personnel.

#### 1b: Collaboration with local hospitals

All the local centers made collaboration agreements with their local hospital, but the collaboration was to some extent hampered by institutional factors. For example, the catchment areas of the local hospitals also include municipalities that were not part of the trial. This limited the number of eligible patients at the hospitals and, consequently, hospital staff acquired limited experience with the service. Further, the GPs, other municipal healthcare services and the hospitals all have different financing systems, which affects their incentives to collaborate and invest in the development of the service. Different informational systems, and associated problems sharing information about patients, were also an often-mentioned challenge.

#### 1c: The GP service

The NDH decided that the patients’ GPs were to be involved in both the design of the telemedicine-based service and in the follow-up of patients in the trial. This was to ensure involvement from the GP, as the experience from a preceding trial was that GPs were not sufficiently involved in the telemedicine follow-up of their patients [24, 25]. The GP service in Norway is part of the municipal healthcare service, but most GPs are private practitioners. Most GP offices are relatively small – in 2021, the average GP office had 3,7 GPs, and there is little capacity at each office to work on service innovation [26]. There is also a low degree of integration between the GP service and other municipal healthcare services. These characteristics made it challenging for the local centers to inform the GPs about the trial and get them onboard.

Everyone who is registered as a resident in a Norwegian municipality is entitled to a regular GP, with the intention that they see the same doctor every time they see their GP. Many elderlies or chronic care patients see their GP for regular check-ups. This practice might work against the trial goal of telemedicine-based follow-up of reducing the use of GP services.

#### 1d: The Covid-19 pandemic

The telemedicine trial was about half-way when the Covid-19 pandemic struck in March 2020. Almost overnight, the provision of remote health services skyrocketed [26]. The pandemic also changed the attitude towards telemedicine follow-up as provided through the trial. The trial’s target group largely included individuals who are at a higher risk of complications if infected by the corona virus. The participating municipalities quickly realized that telemedicine follow-up enabled and facilitated follow-up of patients in this risk group while minimizing the risk of spreading the virus. The patients themselves also appreciated the possibility to be followed-up by health personnel without physical contact.

Due to the pandemic, the awareness of the potential of telemedicine follow-up also increased among health personnel in other parts of the healthcare system. Yet, the practical collaboration around the service was challenged because of the strain that was put on the entire healthcare sector during the pandemic. Local center managers could not focus on service development as planned because of other more pressing work tasks. Thus, while the pandemic provided a boost in the attitudes towards telemedicine, both among patients and health personnel, it also affected service development and collaboration adversely.

Follow-up service nurses and local center managers also described that the content of the follow-up changed during the pandemic. Follow-up service nurses reported that they spent more time talking on the phone with patients than before. Nurses and center managers also reported that to some extent the type of patients who agreed to participate in the trial changed. Some patients had earlier declined the invitation to participate because they experienced the randomization process as a mental strain. During the pandemic, the share of participants with cancer and psychiatric diagnosis, who had previously often declined the invitation to participate in the trial, increased.

### 2: Implementation

In this section, we describe the practical implementation of the telemedicine-based intervention in the local projects, and the variation between the projects.

#### 2a: Organization of the follow-up service and integration with other services

The local centers chose to organize and locate the follow-up service in different ways. Some of the follow-up services were co-located with the HCS unit enabling close cooperation, which simplified identification of eligible patients and coordination of services. Yet, it sometimes resulted in follow-up service nurses performing tasks for the HCS. Other local centers located the follow-up service at the emergency room. An advantage was that it connected the service to a strong professional environment. On the other hand, it involved a greater distance to other municipal services, increasing the barrier for cooperation and interaction. One local center organized the follow-up service as an inter-municipal follow-up service, with the goal to increase the scope of potential patients and professionalize the service within the region, with the disadvantage of a weaker connection to the municipal HCS. One local center, which covered multiple municipalities in a region, chose to try different organizations simultaneously, including both inter-municipal follow-up services and follow-up by a primary healthcare team nurse at the GP office. The organization of the follow-up services mainly remained constant throughout the trial, although some centers made minor adjustments. This suggests that the service can be organized in different ways, allowing for adaptations to the local context.

Telemedicine follow-up was a new service for all but two local centers that previously participated in telemedicine trials [24, 27]. Consequently, the local project managements spent considerable resources on informational meetings with other healthcare services providers in the initial phase of the trial. The purpose of these meetings was threefold: to increase the knowledge of telemedicine follow-up, to establish collaboration between the follow-up service and other service providers around patients, and to identify and recruit patients to the trial. Early in the trial, local center managers expressed that it was essential to regularly schedule meetings at the hospital or with the GPs, to remind them about the new service.

During the trial, the follow-up services identified useful ways to improve the collaboration with other healthcare services and their understanding of the telemedicine service. For example, the local centers employed GPs in part-time positions in the management team to help develop the service in a way that was beneficial for the GP service. They also improved communication with other local GPs, because they “spoke the same language” and included patients from their own patient lists in the trial. Similarly, the local centers employed nurses at the local hospitals, who were responsible for identifying eligible patients at the hospital and to raise awareness of the service at the hospitals. Physical organization closer to municipal HCS was also perceived useful to establish collaboration.

Towards the end of the trial, all local center managers expressed that the understanding of the telemedicine service and its potential benefits had improved considerably among collaborating healthcare service providers. One interpretation is that introducing a new service takes time, and that it is important for other healthcare providers to experience the potential benefits of the new service before they adapt their own follow-up. Furthermore, in a hectic work environment, dedicated time and resources are essential for successful implementation of a new service.

#### 2b: Inclusion of patients

The NDH defined inclusion criteria for the trial, but some of the local centers defined additional inclusion criteria. Some centers focused exclusively on certain diagnoses, such as heart or lung diseases, while others adapted a more flexible approach in assessing potential patients as the trial progressed. During the trial, all local centers adjusted the eligibility assessment from emphasizing the patients’ diagnosis to taking a more holistic approach when evaluating patients’ suitability and potential benefits of the service. Further, some local centers chose to start including cancer patients and patients with mental illness, not necessarily to reduce their use of other healthcare services but rather to provide better care and support. The local centers also experienced that it was essential that patients were motivated for the telemedicine follow-up, to ensure compliance and downstream benefits.

The timing of inclusion was a returning theme in interviews and workshops. The follow-up service nurses experienced that including the patient at the right time in his or her course of illness could be decisive for the extent to which the service could provide benefits in terms of better health and reduced use of healthcare services. Follow-up service nurses and local center managers experienced that patients who would have been allocated HCS, had telemedicine follow-up not been available, had the greatest potential for experiencing postponed need for healthcare services in the short run. Allocating telemedicine follow-up to patients who were either too healthy or too ill tended to decrease the potential benefits of follow-up in terms of reduced or postponed need for healthcare services.

The GP would evaluate the eligibility and approve participation in this trial for each patient. During the trial, however, the responsibility of evaluating eligibility and approving patients for the trial shifted to the nurses at the follow-up service due to several factors. First, this approach was more in line with allocation of other municipal health and care services. Second, the follow-up service nurses had first-hand experience with the service and thereby a better understanding of which patients would benefit from the service. Third, GPs in Norway experience a heavy workload, and thus shifting the responsibility decreased GPs’ use of time and accelerated initiation of follow-up. Experiences from the trial suggested that it was most valuable if the GP contributed to the development of the patient’s individual treatment plan.

#### 2c: Follow-up of patients

The frequency of follow-up varied between local centers and depended on the patients’ health status. Most patients performed medical measurements daily, some less frequently. Most centers provided follow-up Monday to Friday, while one center only provided follow-up once a week. Local center managers and follow-up service nurses seemed to think that follow-up once a week was sufficient, and neither the patient interviews nor the effectiveness analysis found clear evidence supporting that the patients at this center experienced less favorable outcomes in terms of user satisfaction, health status or resource use than patients at other centers [20]. All follow-up services made routine follow-up phone calls to patients. Early in the trial, some follow-up services called the patients every second week, but the frequency of these calls decreased considerably during the trial period, to every second or third month. All centers also regularly evaluated the patients’ need for adjustments in or termination of follow-up.

In interviews, patients who performed daily measurements argued that it was easier to perform measurements every day, rather than to remember to perform measurements on a specific day. Health personnel were divided in their opinions of the frequency of measurements. Some indicated that even though daily measurements might not be necessary from a medical point of view, the high frequency might still be acceptable if it does not incur extra costs to the healthcare service. Others, mainly GPs, argued that the high follow-up frequency generated unnecessary data, and that it could lead to an unhealthy disease focus among patients. Evidence from the trial suggests that patients differ in their needs and preferences and that some degree of personalization is valuable for the patients. A small minority of patients in the trial experienced increased disease focus and anxiety in response to telemedicine follow-up, for which the frequency of follow-up was adjusted or terminated.

In practice, telemedicine follow-up meant that patients would make measurements using home-monitoring devices, answer questions regarding their health status, or both. The local centers chose different approaches to these components. One center had standardized diagnosis-specific follow-up kits, containing both home-monitoring devices and a standardized set of questions. Other centers mainly relied on the results from home-monitoring devices, while others mainly relied on information from answered questions. One common argument for using results from home-monitoring devices was that they provided objective measures of the patient’s health status. The use of patient questionnaires, on the other hand, was motivated by cost containment, and that simple health-related questions were often sufficient to detect deteriorations in health status.

### 3: Mechanisms

In this section, we describe some of the mechanisms through which the telemedicine-based intervention brings about change. The first part describes patients’ interaction with and responses to the service. The second part describes the perspective of health personnel.

#### 3a: Patient interaction with the service

**3a1: User interaction** Most patients mastered the equipment well, even though many of them lacked previous experience with computer tablets and other technological devices. Patients reported that the follow-up application was easy to use, and problems were mainly related to software upgrades and log-in issues. Some of the local centers locked the tablets so that only the follow-up application was available for the patients. None of the patients we interviewed experienced follow-up as time consuming. Several patients described that the measurements were a natural part of their daily routine:

*“It has become a pleasure for me to make these measurements. It’s relaxing and it has become a habit that I’m happy to have.” (Patient 1)*.

Follow-up service nurses described, however, that some patients, typically those who were younger and healthier, had terminated follow-up because they felt the time-cost did not outweigh the perceived benefits.

Patients differed in their use of the information that was stored on the computer tablet. Some patients actively reviewed the measurement trends, while others left monitoring and interpretation of the results to the follow-up service. There were also examples of next of kin who reviewed the measurement trends closely. Patients who more actively reviewed their trends gave the impression that they learned more about their symptoms and to better cope with their health condition.

Patients described their reactions to measurement results in different ways. Most patients expressed that performing regular measurements led to an increased sense of safety. Some patients also described that taking the measurements could be encouraging, especially if the results were better than they expected. Abnormal measurements, however, led to feelings of insecurity and anxiety among some patients.

*“… and it’s like when I see the measurement and “oh, this is not good”, then I don’t like it. The next day it could be “wow, today I am doing well”, so it’s a bit of both. But I’d like to say that it’s comforting to know that I can call them [the follow-up service] or they can call me.” (Patient 2)*.

Follow-up nurses reported that a small number of patients terminated the service due to stress and anxiety related to the regular measurements.

The intention of the individual treatment plan was to increase user involvement, and to enable patients to immediately react to changes in their health status. However, patient interviews gave little support to the hypothesis that individual treatment plans improved patients’ sense of user involvement. Follow-up service nurses and local center managers, on the other hand, stressed that the individual treatment plan helped patients act on symptoms of exacerbation faster, for example by taking antibiotics or seeking professional help. The contrasting results may be a consequence of selection of patients for the interviews. Patients who consented to being interviewed may have been healthier and therefore less dependent on the individual treatment plan than the average patient in the trial.

**3a2: User satisfaction** The effectiveness analysis of the trial found that 12 months after inclusion, participants in the intervention group were significantly more satisfied with the follow-up of their health compared to the control group [21]. Semi-structured interviews with patients largely confirmed these findings. Patients reported that the follow-up service nurses were attentive and genuine, and that they felt a personal bond with the nurses. There was a rather large degree of personalization of the intervention depending on patient needs and preferences, and this generated value for the patients. Patients who initially experienced that the follow-up was too frequent or invasive, made necessary adaptations in consultation with the nurse.

Almost all interviewed patients reported that they felt safer with telemedicine follow-up. Several elements contributed to this, including users gaining knowledge about their own illness and health, that a nurse was monitoring their measurements, and that they could contact the follow-up service if needed.

Among the patients, there was broad agreement that the intervention contributed to increased knowledge about one’s illness and health. The follow-up service nurses worked actively to guide and motivate patients, which contributed to improved understanding of their condition and symptoms. A nurse said that even if some of the patients experienced deteriorations, their ability to perceive their body’s signals and symptoms improved, such that they became better at taking care of their own health in the longer run. The findings of increased understanding of one’s illness was also confirmed in the effectiveness analysis [21].

We also studied whether the telemedicine intervention affected the everyday life of the patients in a broader perspective. In interviews, most patients reported that the telemedicine follow-up did not affect their lives, which were in accordance with our previous quantitative analyses of survey data [21]. Some patients were too sick to leave their homes for trips or social activities, although the telemedicine follow-up had improved their feeling of safety. Other patients, who were healthier, were not impaired by their disease and lived their life like before. We were, however, given examples of patients who dared to live a more active life, for example by travelling abroad or to their cabin, because they now had better tools to understand their illness and symptoms.

One of the aims with telemedicine-based follow-up was to reduce the use of other healthcare services, for which our results from the effectiveness analysis was mixed [21]. While most of the interviewed patients did not experience significant changes in their use of healthcare services, some gave examples of changes. One patient reported that she previously received HCS daily, while she now only needed assistance with showering. A follow-up service nurse described a cancer patient who did not want her neighbours to see the home-nurses car in her driveway and was pleased when she could receive telemedicine follow-up. On the other hand, project staff reported in interviews and workshops that some patients were unwilling to reduce their use of HCS even though this would be medically justified.

Most patients went to regular check-ups with their GP and specialist doctor at the hospital, and for most of them the frequency of these consultations did not change in response to the telemedicine follow-up. There were exceptions to this: some patients experienced seeing their GP less often, while others experienced more frequent consultations because either the patient or the GP wanted an extra consultation in response to abnormal measurements.

Although not all GPs were interested in the home measurements, many patients and GPs stressed that telemedicine follow-up improved the quality of consultations because of the patients’ increased understanding of their symptoms. Patients and some GPs reported that it was useful to review the home measurements together, and that the increased patient involvement led to higher quality discussions in the consultations.

It was difficult for the patients to evaluate the impact of the telemedicine follow-up on unplanned hospital admissions. A couple of patients were, however, certain that admissions had been avoided due to the telemedicine follow-up since deteriorations had been noted and handled earlier.

Taken together, it seems that the greatest value for the patients comes from the contact with the follow-up service, rather than from the monitoring of vital signs. Due to the complexity of this trial, it has not been possible to quantitatively investigate further which parts of the intervention bring the greatest value to patients.

#### 3b: Healthcare staff responses to the service

**3b1: Follow-up service responses** All measurements were sent to the follow-up service. In case of an abnormal measurement, the nurse would call or write a message to the patient. Often, the patient was asked to make a new measurement, and sometimes it turned out that the initial measurement had been faulty.

Several informants pointed out that operating the follow-up service requires a special skill set. Follow-up service nurses must be able to interpret the developments in reported measurements, while being able to use digital communication with patients and to identify changes in their health status based on the tone of the patient’s voice or a video call. Thus, the competence of the follow-up service nurse is central to the service.

*“… [the follow-up service nurses] must be the kind of person who are comfortable making decisions even if they haven’t had the chance to see and touch the patient. They need to be good at putting together the digital information.” (Local center manager 1)*.

**3b2: Municipal home-based care services (HCS)** From interviews with both users and health professionals, we learned how the telemedicine invention can contribute to changed use of HCS.

In the first half of the trial period, some centers experienced challenges in identifying users for whom it is possible to reduce the use of HCS. Local center staff and representatives for HCS described that some patients who already had HCS were unwilling to give up these services. Therefore, it was difficult to reduce resource use in HCS in response to the telemedicine follow-up.

“*…this far it’s been a service in addition to other services, so it’s not like they have lost anything in a way.” (Local center manager 1)*.

Subsequently the municipalities shifted their practice when municipal services were allocated to patients. By stating that telemedicine follow-up should be the first municipal HCS to be allocated when a need for HCS is identified, the municipalities hoped to postpone the need for more comprehensive HCS for some of their inhabitants.

In subsequent interviews, local center managers also expressed that it was possible to reduce the use of HCS for more users, partly because of closer cooperation with the home service unit, and thereby better knowledge of the intervention.

**3b3: GP responses** GPs were divided in their opinions about telemedicine follow-up from the start of the trial. Some embraced the new service and its potential for better follow-up of patients with chronic disease and reduced time use. Others were more reluctant, and feared increased time use, e.g., because of patients demanding extra consultations due to abnormal measurements.

*“I would like to have access to measurements and figures, but as you know, we have a lot to deal with, so this means there will be one more service involved. It depends on the number of patients and inquiries that may arise.” (GP 1)*.

In interviews and workshops, GPs expressed that they were reluctant to change their follow-up of patients until they get acquainted to telemedicine follow-up. Follow-up service nurses reported that GPs’ attitudes towards the service became more positive during the trial period, as the GPs gained experience with the intervention. This suggests that it takes time before GPs change their follow-up of patients who are telemedicine users.

**Specialized healthcare** The local centers experienced that it was difficult to actively involve specialized healthcare in the trial. In practice, specialized healthcare mainly contributed by identifying and recruiting patients to the trial. This was typically done by a nurse who was paid by the project. Patients were mainly identified during in-patient stays, but some were recruited through out-patient consultations.

Specialist doctors were not significantly involved. In some complex cases, specialist doctors were involved in developing the individual treatment plan. Specialist doctors could not access the patients’ measurements, but patients could bring their computer tablets to the hospital. The general impression from patient interviews was that specialist doctors were generally not interested in the measurements. On the one hand, it raises a potential for closer integration of healthcare services. The lack of interest may, on the other hand, serve as an obstacle for effective integration of services, and for the utilization of the full potential of the telemedicine-based service.

Many patients had regular checkups with a specialist at the hospital, partly regulated by national guidelines. Unless national guidelines are adjusted in accordance with telemedicine implementation, we do not expect the use of out-patient services to change significantly in response to the telemedicine-based service.

## Discussion

The purpose of this study has been to contribute to a broader understanding of the implementation and the outcomes of a telemedicine-based intervention in primary healthcare in Norway [20] by interviewing a considerable number of patients participating in a randomized controlled trial, their next of kin, and health personnel about their experiences. The results reported in this article, complement an effectiveness analysis of the trial documented by Sten-Gahmberg et al. [21]. We found the chosen process evaluation framework [17] emphasizing the relations between context, implementation, and mechanisms to work well as an analytical tool to bring out important aspects regarding the trial. The framework made it possible to highlight the anticipated impact of different actors on the trial results. For example, it became clear what role GPs play and that they are an important actor who likely must change their way of working to make telemedicine-based follow-up of patients a cost-effective service.

As part of the telemedicine follow-up, persons with chronic conditions would use a computer tablet and relevant home-monitoring devices to perform measurements and answer questions that were automatically transferred to a local follow-up service. The technological solutions informed the patient and the follow-up service when the measurement results deviated from the patient’s normal values. The nurse responded to abnormal results and considered relevant actions in consultation with the patient. The service is complex, yet flexible, both with respect to the heterogeneity in the target group, the design and implementation of the service, and its interaction with other healthcare providers.

Our effectiveness analysis shows that the telemedicine-based intervention contributed to increased patient satisfaction, security, and self-efficacy compared to usual care [21]. Telemedicine users avoided a deterioration in their health in the first year after inclusion reflected in a significant gain in quality-adjusted life-years (QALY) comparable to that of other similar services [28]. Additionally, telemedicine users reduced their use of HCS, but did not change their use of specialist healthcare services. The use of GP services increased among the telemedicine users, mainly because of an increase in communication between the GP and other healthcare providers about patients. Our cost-benefit analysis shows that the costs of providing the intervention likely exceed benefits that can be monetized within a cost-benefit framework [21]. However, the telemedicine-based intervention contributes to considerable non-monetized effects such as safety and self-efficacy, also found in similar studies [29].

In line with the findings of our previously published effectiveness analysis, this interview study finds that patients were mainly satisfied with the telemedicine-based follow-up, a common finding in studies on patient experiences with telemedicine-based services [30–32]. Most patients expressed that the follow-up contributed to an increased sense of safety and improved understanding of their own symptoms and health. These findings are largely in line with previous research [3, 33]. Still, our impression from our interviews is that some patients were significantly less interested and involved in the monitoring of their health statistics and left this task solely to the follow-up service. This may hamper the impact of follow-up and raises the question of patients’ ability to be more actively involved in the follow-up, and how. Little is known about how patients perceive digital health information and how their level of health literacy play into this [34, 35]. This calls for further research. It is also important to note that the telemedicine-based service does not suit all patients. A small number of patients terminated follow-up because of increased anxiety and disease focus, and some because their perceived benefit did not exceed their perceived cost of follow-up.

The effectiveness analysis showed mixed results on use of other healthcare services [21]. There was a reduction in the use of HCS, but no change in the use of specialized healthcare services. The latter is a relatively common finding in the literature [12–14, 33]. The present qualitative analysis shows that a partial explanation for this may be that it takes time to build a new service, to raise awareness and to integrate it in the existing healthcare system. Future research should evaluate the longer-term impact of telemedicine-based follow-up on the use of other healthcare services, especially specialized healthcare services.

Our analysis sheds light on the importance of using dedicated resources to develop and implement a new telemedicine-based service, and to involve other healthcare services. Within the trial setting, this was possible due to financing from the NDH. However, it raises the question of how successful the implementation would be outside a trial setting without dedicated resources. This is also relevant considering that the telemedicine-based service has proven to be a highly flexible service that can be adjusted to many different patient groups, personal needs, and organizational landscapes.

While this flexibility constitutes an advantage, it may also be a drawback. The flexibility makes it more difficult to quality control, cost-contain, and evaluate. One can question how the service will develop over time when it is no longer closely monitored by researchers and dedicated project personnel, or when it is implemented in new municipalities. In Norway, the NDH will assist municipalities in implementing the service, by providing guidance and financial support through a national program for welfare technology [36, 37].

Our results suggest that telemedicine-based interventions have potential to become an important part of future primary healthcare if organized effectively. Implementation in clinical practice likely requires a trial-and-error approach, as this field is still developing. Such implementation should be accompanied by research-based process evaluations preferably based on mixed-methods approaches to reach a better understanding of the complexity related to telemedicine-based interventions.

This study takes a novel approach as it is conducted as part of a pragmatic randomized controlled trial. Such a setting has several benefits. First, it gives a broader understanding of the intervention, and the results reported in this study give meaning and interpretation to the effects identified in the effectiveness analysis, and the lack thereof. Second, the use of data from semi-structured interviews with patients, next of kin, and health personnel allows us to provide a more complete picture of the context of the service, its implementation, and mechanisms through which the service may lead to effective change or meet barriers. At the same time, the heterogeneity and flexibility in the service made it challenging to pin down which parts of the intervention contributed to effective change. Nonetheless, the pragmatic nature of the trial has contributed to useful insights and adjustments in the telemedicine-based service underway, in ways that a rigorous randomized controlled trial would not allow for.

### Limitations

There are, nonetheless, limitations to the study. First, the interviewees were recruited to the study with the help of the local center management. In the case of patients, there are indications that the patients that agreed to be interviewed are healthier than the average patient in the trial, which may affect our interpretation of the intervention. It is also possible that local center managers contacted patients with success stories to highlight the positive sides of the service. GPs are known to be reluctant to taking on extra tasks, and thus one could imagine that GPs who accepted to be interviewed were more likely to have strong opinions about the service.

## Conclusion

This study has shown that the implementation of a telemedicine-based service in primary healthcare is a complex process that is sensitive to contextual factors and requires time and dedicated resources to ensure successful implementation. We find that patients are mainly satisfied with the service and point at factors that may have contributed to the absence of significant reduction in the use of GP services and specialized healthcare services. This study contributes to the understanding of how the implementation of a telemedicine-based service intervention can be done in practice, and how a process evaluation can give additional insight to the evaluation of randomized controlled trials.

### Electronic supplementary material

Below is the link to the electronic supplementary material.

**Additional file 1:** Interview guide: Patients

**Additional file 2:** Interview guide: Patients and next of kin

**Additional file 3:** Interview guide: Local center managers

**Additional file 4:** Interview guide: Staff in the follow-up service

**Additional file 5:** Interview guide: GPs with patients receiving telemedicine-based follow-up

**Additional file 6:** Interview guide: Hospital staff

**Additional file 7:** Interview guide: Home care services

## Data Availability

The data analyzed during the current study are available from the corresponding author on reasonable request.

## Abbreviations

COPD: chronic obstructive pulmonary disease
GP: General practitioner
HCS: Home–based care services
NDH: Norwegian Directorate of Health
QALY: Quality–adjusted life–years
