# Correction: Experiences with telemedicine-based follow-up of chronic conditions: the views of patients and health personnel enrolled in a pragmatic randomized controlled trial

**DOI:** 10.1186/s12913-024-10890-8

**Published:** 2024-04-08

**Authors:** Susanna Sten-Gahmberg, Kine Pedersen, Ingrid Gaarder Harsheim, Hanna Isabel Loyland, Birgit Abelsen

**Affiliations:** 1 Oslo Economics, Klingenberggata 7, 0161 Oslo, Norway; 2 Present Address: The Finnish Centre for Pensions, FI-00065 Eläketurvakeskus, Finland; 3 Department of Health Management and Health Economics, University of Oslo, Postboks 1089, 0317 Blindern, Oslo Norway; 4 Department of Community Health, UiT - The Arctic University of Norway, 9037 Tromsø, Norway

**Correction to: BMC Health Services Research (2023) 24:341**

10.1186/s12913-024-10732-7

In this article, affiliation 2 (Present address: The Finnish Centre for Pensions, Eläketurvakeskus FI-00065, Finland) was incorrectly assigned to Kine Pedersen, Ingrid Gaarder Harsheim and Hanna Isabel Løyland, instead of affiliation 1 (Oslo Economics, Klingenberggata 7, Oslo 0161, Norway). These errors were caused by a typesetting mistake.

The errors are corrected in the author list of this Correction article and the original article has been updated. The publisher apologises to the authors and readers for the inconvenience caused by this error.

