# Supplementary material for: Experiences with telemedicine-based follow-up of chronic conditions: the views of patients and health personnel enrolled in a pragmatic randomized controlled trial
Source: BMC Health Serv Res. 2024 Mar 14;24:341. doi: 10.1186/s12913-024-10732-7 (PMC10941467; doi:10.1186/s12913-024-10732-7)
Supplement: Supplementary file 1 — Additional file 1: Interview guide: Patients [file 12913_2024_10732_MOESM1_ESM.docx]

# Additional file 1, Interview guide: Patients

**Interview guide for users of telemedicine-based follow-up**

The University of Oslo, Oslo Economics, and the Norwegian Centre for Rural Medicine are conducting a research project on behalf of the Norwegian Directorate of Health to study the effects of telemedicine-based follow-up. As part of this project, we would like to conduct interviews with individuals using telemedicine-based follow-up to learn about their experiences. Below, you will find some questions we would like to ask you. We may not necessarily ask all the questions during the interview, and you are also welcome to bring up other topics that you believe are relevant.

**BACKGROUND**

- How did the recruitment process for telemedicine-based follow-up take place for you?
- What were your initial thoughts when you first learned about telemedicine-based follow-up?

**FOLLOW-UP THROUGH TELEMEDICINE-BASED FOLLOW-UP**

- In what ways are you monitored through telemedicine-based follow-up?
  - What equipment have you received?
  - How often do you use the equipment?
- Have you changed the way you use the equipment since you started, and if so, how?
- Can you describe your contact with the follow-up service?
  - How often do you interact with the follow-up service?
  - Do you receive answers to your questions?

**TELEMEDICINE-BASED FOLLOW-UP IN DAILY LIFE**

- In what ways has telemedicine-based follow-up affected your daily life?
  - Your daily planning
  - Your activities inside and outside the home
  - Your sense of security
- Are your next of kin involved in the telemedicine-based follow-up you receive?
  - What are their thoughts on this service?

**HEALTH STATUS**

- Has telemedicine-based follow-up influenced your knowledge about your health condition and how you manage your illness?
- Has telemedicine-based follow-up affected your health status?
  - The stability of your health condition
  - Your use of medications?

**USE OF HEALTH SERVICES**

- Has telemedicine-based follow-up influenced your contact with your general practitioner and other parts of the healthcare system (e.g., home care services)?
- Do you feel that telemedicine-based follow-up replaces some of your needs for other health services, or is it in addition to other services?

**EXPERIENCES WITH TELEMEDICINE-BASED FOLLOW-UP**

- Which aspects of the monitoring via the tablet are you most satisfied with?
- Has telemedicine-based follow-up met your expectations?
- What are the most important positive experiences?
- What are the most important negative experiences?
- Is there anything else you would like to convey?
