# Supplementary material for: Experiences with telemedicine-based follow-up of chronic conditions: the views of patients and health personnel enrolled in a pragmatic randomized controlled trial
Source: BMC Health Serv Res. 2024 Mar 14;24:341. doi: 10.1186/s12913-024-10732-7 (PMC10941467; doi:10.1186/s12913-024-10732-7)
Supplement: Supplementary file 3 — Additional file 3: Interview guide: Local center managers [file 12913_2024_10732_MOESM3_ESM.docx]

# Additional file 3, Interview guide: Local Center Managers

**Interview guide for interview with local center managers for telemedicine-based follow-up**

The University of Oslo, Oslo Economics, and the Norwegian Centre for Rural Medicine are conducting a research project on behalf of the Norwegian Directorate of Health to study the effects of telemedicine-based follow-up. As part of this project, we would like to conduct interviews with the municipal project manager for telemedicine-based follow-up (and any other relevant individuals in the project) to learn about their experiences. Below, you will find some questions we would like to ask you. We may not necessarily ask all the questions during the interview, and you are also welcome to bring up other topics that you believe are relevant.

The follow-up service is differently organized and has different names in the various participating municipalities. Among other terms, follow-up service, Health Watch, and Telemedical Central (TMC) are used. For simplicity, we use "follow-up service" in this interview guide.

**ORGANIZATION OF THE SERVICE**

- Can you tell us about the organization of the service?
  - Have there been any changes in the organization of the follow-up service?
  - Could the follow-up service be organized more efficiently than it is today?
- Which patient groups receive telemedicine-based follow-up?
  - How are the patients selected?
- At what stage in the course of the disease should patients be included? *[Included only in 2020]*
- How was the recruitment of staff for the follow-up service?
  - Do you have any thoughts on why it was easy or difficult (working hours, workload, tasks)?

**COLLABORATION AND INTERACTION**

- How does collaboration on telemedicine-based follow-up work between different parts of the healthcare service?
  - Which aspects of collaboration work well?
  - What are the most important collaboration challenges?
- How has telemedicine-based follow-up affected the division of labor among the involved parties?
  - GP, home care, follow-up service, patients, relatives?
- How is the content of the self-treatment plan determined? What should be measured? *[Included only in 2020]*
- Do have experience with terminating telemedicine-based follow-up for some patients? What was the reason? *[Included only in 2020]*

**BENEFITS (FOR SERVICE LOCATION, PATIENT, AND SOCIETY)**

- What are the most important benefits of telemedicine-based follow-up for the municipal health service, general practitioner service, and specialized health service, as you see it?
- Can you describe your motivation for continuing to offer telemedicine-based follow-up to residents in your municipality/district?
- What difference do you perceive telemedicine-based follow-up making for the patients?
  - Which components of telemedicine-based follow-up do you find most beneficial for the patient?
- Are there any benefits associated with telemedicine-based follow-up that could be realized more efficiently in other ways?
- What direct or indirect costs do you see associated with telemedicine-based follow-up (for various parts of the healthcare service, for patients, and others)?

**SUCCESS CRITERIA AND CHALLENGES**

- What were the most important challenges before the trial started?
- What challenges have been the most important since the trial started?
- What are the key success criteria for telemedicine-based follow-up to work effectively? *[Included only in 2020]*
- If you were to start the trial again, is there anything you would do differently? *[Included only in 2020]*
- What advice do you have for new municipalities embarking on telemedicine-based follow-up? *[Included only in 2020]*

**NATIONAL CONTEXT**

- Do you think telemedicine-based follow-up is a good measure to meet the needs for health and care services in the aging population?
- What do you think will be the most important benefits of a national expansion of telemedicine-based follow-up?
- What do you think will be the most important challenges of a national expansion of telemedicine-based follow-up?
- What are your thoughts on the differences in organization between projects? *[Included only in 2020]*

**FOLLOW-UP OF PATIENTS IN THE CONTROL GROUP**

- How do patients in the control group feel about being in the control group?
- What do you know about the follow-up of patients in the control group? Do they receive services they would not have received if they were not in the control group?
- Are they participating in other trial projects?
- Is there anything else you would like to convey?
