# Supplementary material for: Experiences with telemedicine-based follow-up of chronic conditions: the views of patients and health personnel enrolled in a pragmatic randomized controlled trial
Source: BMC Health Serv Res. 2024 Mar 14;24:341. doi: 10.1186/s12913-024-10732-7 (PMC10941467; doi:10.1186/s12913-024-10732-7)
Supplement: Supplementary file 4 — Additional file 4: Interview guide: Staff in the follow-up service [file 12913_2024_10732_MOESM4_ESM.docx]

# Additional file 4, Interview guide: Local staff working in the Follow-up service

**Interview Guide for Interviews with Individuals Working on Follow-up of Users of Telemedical Remote Monitoring**

The University of Oslo, Oslo Economics, and the Norwegian Centre for Rural Medicine, on behalf of the Directorate of Health, are conducting a research project to study the effects of telemedical telemedicine-based follow-up. In connection with this project, we would like to conduct interviews with individuals who are involved in the follow-up of users of telemedical telemedicine-based follow-up to learn about their experiences. Below, you will find some questions that we would like to ask you. We may not necessarily ask all the questions in the interview, and you are also welcome to address other topics that you believe are relevant.

The follow-up service is differently organized and has different names in the various participating municipalities. Among other terms, follow-up service, Health Watch, and Telemedical Central (TMC) are used. For simplicity, we use "follow-up service" in this interview guide.

**ORGANIZATION**

- Which patient groups receive telemedicine-based follow-up?
  - How are individual patients selected?
  - At what stage in the course of the disease should patients be included? *[Included only in 2020]*
  - What experiences do you have with different patient groups? Are there some more suitable for telemedicine-based follow-up than others? Are there some who need closer follow-up than others?
- What components can be included in your follow-up?
  - Measurements, questions, messaging function, video, tips/encouragements/advice, other?
- How does the organization of the follow-up service work?
  - What are the opening hours of the follow-up service?
  - Where is the follow-up service located?
  - Who works in the follow-up service? How many?
  - Have there been any changes in the organization of the follow-up service since its inception?
- How do you work in the follow-up service on a daily basis?
  - What types of inquiries do you receive?
  - What problems do you encounter, and how are they resolved?
  - Do you manage to get everything done in a day?
  - To what extent do you involve others (home services, general practitioner, hospital, etc.) in the follow-up?
- Could the follow-up service be organized more effectively than it is today?
- Do have experiences with terminating telemedicine-based follow-up for some patients? What was the reason? *[Included only in 2020]*

**COOPERATION AND INTERACTION**

- How do you collaborate with other parts of the healthcare service?
  - Which parts of the collaboration work well?
  - Where are the most important collaboration challenges?
- Do you feel that telemedicine-based follow-up has influenced the division of labor among the involved parties?
  - General practitioner, home nursing, the follow-up service, patients, family?

**BENEFITS AND COSTS (FOR SERVICE PROVIDER, PATIENT, AND SOCIETY)**

- What are the most important benefits of telemedicine-based follow-up for different parts of the healthcare service?
- What are the most important benefits of telemedicine-based follow-up for the patients?
  - Which components of telemedicine-based follow-up do you find most useful for the patient?
- What costs are associated with telemedicine-based follow-up for different parts of the healthcare service?
- Does telemedicine-based follow-up incur costs or disadvantages for the patients?

**SUCCESS CRITERIA AND CHALLENGES**

- What are the most important success criteria for telemedicine-based follow-up to function well locally?
- What are the most important success criteria for telemedicine-based follow-up to function well in a potential national expansion of the initiative?
- What are the biggest challenges related to telemedicine-based follow-up in your municipality?
- What are the biggest challenges related to telemedicine-based follow-up in a potential national expansion of the initiative?
- If you were to start the trial again, is there anything you would do differently? *[Included only in 2020]*

**NATIONAL CONTEXT**

- Do you/they believe that the trial of telemedicine-based follow-up is a good measure to meet the needs for health and care services in the aging population?
- What are your thoughts on the differences in organization between projects? *[Included only in 2020]*
- Is there anything else you would like to convey?
