# Supplementary material for: Experiences with telemedicine-based follow-up of chronic conditions: the views of patients and health personnel enrolled in a pragmatic randomized controlled trial
Source: BMC Health Serv Res. 2024 Mar 14;24:341. doi: 10.1186/s12913-024-10732-7 (PMC10941467; doi:10.1186/s12913-024-10732-7)
Supplement: Supplementary file 5 — Additional file 5: Interview guide: GPs with patients receiving telemedicine-based follow-up [file 12913_2024_10732_MOESM5_ESM.docx]

# Additional file 5, Interview guide: GP with patients receiving telemedicine-based follow-up

**Interview guide for interviews with general practitioners (GPs) whose patients receive telemedicine-based follow-up**

The University of Oslo, Oslo Economics, and the Norwegian Centre for Rural Medicine are conducting a research project on behalf of the Norwegian Directorate of Health to study the effects of telemedicine-based follow-up. As part of this project, we would like to conduct interviews with general practitioners who have patients receiving telemedicine-based follow-up to learn about their experiences. Below, you will find some questions we would like to ask you. We may not necessarily ask all the questions during the interview, and you are also welcome to bring up other topics that you believe are relevant.

The follow-up service is differently organized and has different names in the various participating municipalities. Among other terms, follow-up service, Health Watch, and Telemedical Central (TMC) are used. For simplicity, we use "follow-up service" in this interview guide.

**INCLUSION OF PATIENTS**

- How do you experience the inclusion/exclusion criteria for the trial of telemedicine-based follow-up?
  - Do the inclusion/exclusion criteria lead to the right individuals being included in the trial?
  - Are there patient groups that are overlooked but would have been well-suited?
- Do you think it is appropriate for you as a GP to assess patients for telemedicine-based follow-up?
- How many patients on your list do you believe are suitable for telemedicine-based follow-up?
- How many patients on your list are participating in the trial?
- Have you considered proposed patients unsuitable for participation? What was your assessment?
- Do you have patients who have ended telemedicine-based follow-up? Why was the follow-up terminated?
- How do you work on recruitment, and how much time have you spent on recruitment?
- How is the content of the self-treatment plan determined? What should be measured? *[Included only in 2020]*

**PATIENT FOLLOW-UP**

- How do you access patient measurements/responses/other information from telemedicine-based follow-up?
  - How do you utilize the measurements?
- Has telemedicine-based follow-up changed your follow-up of patients receiving it?
- How do you follow up patients included in the control group?

**COLLABORATION AND INTERACTION**

- How does collaboration with other parts of the healthcare system related to telemedicine-based follow-up work?
  - Which aspects of the collaboration work well?
  - What are the most important collaboration challenges?
- How has telemedicine-based follow-up affected the division of labor among the involved parties in the follow-up of individual patients?
  - GP, home care, follow-up service, patients, relatives?

**BENEFITS AND COSTS (FOR GP SERVICE, PATIENT, AND SOCIETY)**

- In what ways does telemedicine-based follow-up affect your work as a GP?
  - How does it affect your tasks?
  - How does it affect your responsibility?
- Can you describe your motivation for continuing to offer telemedicine-based follow-up to your patients?
- What difference do you perceive telemedicine-based follow-up makes for the patients?
  - Which components of telemedicine-based follow-up do you find most beneficial for you as a GP and for the patient?
- Are there any benefits associated with telemedicine-based follow-up that could be realized more efficiently in other ways?
- Does telemedicine-based follow-up incur any costs (direct or indirect) for you as a GP?

**SUCCESS CRITERIA AND CHALLENGES**

- What is essential to involve GPs in telemedicine-based follow-up?
- What challenges do you experience as a GP with telemedicine-based follow-up?
- For whom is telemedicine-based follow-up a good measure? *[Included only in 2020]*

**LOCAL AND NATIONAL CONTEXT** *[Included only in 2019]*

- Do you believe telemedicine-based follow-up is a good measure to meet the needs for health and care services in the aging population?
- What do you think will be the most important benefits of a national expansion of telemedicine-based follow-up?
- What do you think will be the most important challenges of a national expansion of telemedicine-based follow-up?
- Is there anything else you would like to convey?
