# Supplementary material for: Experiences with telemedicine-based follow-up of chronic conditions: the views of patients and health personnel enrolled in a pragmatic randomized controlled trial
Source: BMC Health Serv Res. 2024 Mar 14;24:341. doi: 10.1186/s12913-024-10732-7 (PMC10941467; doi:10.1186/s12913-024-10732-7)
Supplement: Supplementary file 7 — Additional file 7: Interview guide: Home care services [file 12913_2024_10732_MOESM7_ESM.docx]

# Additional file 7, Interview guide: Home Care Services

**Interview Guide: Home Care Services' Experiences with Digital Home Monitoring**

The University of Oslo, Oslo Economics, and the Norwegian Centre for Rural Medicine are conducting a research project on behalf of the Norwegian Directorate of Health to study the effects of telemedicine-based follow-up. As part of this project, we would like to conduct interviews with home care service staff who have experience with telemedicine-based follow-up to learn about their experiences. Below are some questions that we would like to ask you. We may not necessarily ask all the questions during the interview, and you are also welcome to bring up other topics that you believe are relevant.

The follow-up service is differently organized and has different names in the various participating municipalities. Among other terms, follow-up service, Health Watch, and Telemedical Central (TMC) are used. For simplicity, we use "follow-up service" in this interview guide.

**YOUR EXPERIENCE AND KNOWLEDGE OF DIGITAL HOME MONITORING**

- Can you tell us about your position and your knowledge of telemedicine-based follow-up?

**PATIENT INCLUSION**

- How does the staff working at the home care service identify patients suitable for telemedicine-based follow-up?
- What characteristics define patients that staff at the home care service believes may benefit from telemedicine-based follow-up?
- What characteristics define patients who are likely not to benefit from telemedicine-based follow-up?

**PATIENT FOLLOW-UP**

- How does telemedicine-based follow-up affect patient follow-up in the home care service?
- Does telemedicine-based follow-up complement or replace other healthcare services?
- How are the measurements used in home care service?
- Do you feel that the home care service has an appropriate role and responsibility in telemedicine-based follow-up?

**COLLABORATION AND INTERACTION**

- How do you collaborate with the follow-up service and any other healthcare services on telemedicine-based follow-up?

**BENEFITS VS. COSTS**

- What advantages and disadvantages do you think patients experience with telemedicine-based follow-up?
- Do you notice any changes for relatives of patients receiving telemedicine-based follow-up?
- Do you believe that telemedicine-based follow-up helps reduce the need for healthcare services for patients? In what ways, if so?
- How is the home care service affected by patients being offered telemedicine-based follow-up? What services increase, and which decrease?
- Can you think of other consequences of telemedicine-based follow-up for the healthcare service and society?

**SUCCESS CRITERIA AND CHALLENGES**

- For whom is telemedicine-based follow-up a good intervention?
- Could telemedicine-based follow-up be organized differently to achieve better results (related to patient follow-up and more efficient resource utilization in home care)?
- Is there anything else you would like to convey?
